# Lipid emulsion in blood increases extraction of amitriptyline in liposome augmented peritoneal dialysis in rats chronically dosed with amitriptyline: could nanoparticles mitigate the limitations to dialysis in intoxication?

**DOI:** 10.1186/s40635-025-00812-1

**Published:** 2025-09-30

**Authors:** Justin Koh, Matthew Quance, Martyn Harvey, Debra Chalmers, Grant Cave

**Affiliations:** 1 Te Whatu Ora Health New Zealand Te Matau a Maui Hawke’s Bay, Hastings, Hawke’s Bay, New Zealand; 2 Emergency Department, Te Whatu Ora Waikato, Hamilton, New Zealand

**Keywords:** Dialysis, Poisoning, Liposomes, Fat Emulsions, Intravenous

## Abstract

**Background:**

The reach of dialysis in toxicology is limited by two factors, high toxicant volume of distribution and low dialytic extraction of protein bound toxicants in blood. Therapeutic actions for lipid emulsion as antidote are thought involve a “lipid shuttle”, whereby lipid droplets in the circulation “shuttle” lipophilic toxicants with “boarding” in well perfused heart and brain tissue with high toxicant concentrations and “exit” to biologically inert slower equilibrating sites such as muscle or adipose where toxicant concentrations are lower. Such a mechanism raises the conceptual possibility of an extracorporeal “exit” potentially mitigating toxicity through increased drug clearance. In experimental models drug binding nanoparticles in dialysate have been shown to mitigate the problem of blood proteins binding toxicant. We investigated whether the addition of intravenous lipid emulsion would increase extraction of amitriptyline into nanoparticle augmented peritoneal dialysate in rats orally dosed with amitriptyline for 1 week.

**Methods:**

Rats were dosed with amitriptyline in drinking water for a week. On the day of the experiment, anaesthetised rats received either an initial bolus then infusion of lipid emulsion for one hour, or a bolus of saline at the initiation of the experiment equal to the total volume of lipid emulsion given. After a 50 min equilibration period, a 10 min pH gradient nanoparticle augmented peritoneal dialysis dwell was undertaken. Animals were humanely euthanised at the end of the experiment. Blood was sampled 0, 10, 45 and 60 min and peritoneal dialysate was analysed for amitriptyline concentration.

**Results:**

There were no significant differences in baseline physiology, initial amitriptyline blood concentration, nor pulse and blood pressure at any time between groups. Time weighted individual subject mean blood amitriptyline concentrations (median (IQR)); control 104 (87–125) nmol/l, lipid 219 (148–357) nmol/L, *p* = 0.03 and dialysate amitriptyline concentration; control 31(14–52) nmol/L, lipid 105 (62–185) nmol/L, *p* = 0.03 were greater in animals given intravenous lipid emulsion.

**Conclusion:**

These are the first data to our knowledge showing experimental support for the approach of simultaneously decreasing volume of distribution with an intravascular nanoparticle in conjunction with a drug binding particle in dialysate. Further work in this area is warranted.

## Background

Therapeutic actions for lipid emulsion as antidote are thought involve a “lipid shuttle”, whereby lipid droplets in the circulation “shuttle” lipophilic toxicants from well perfused heart and brain tissue to biologically inert slower equilibrating sites such as muscle or adipose [1–3]. To distribute drugs to inert sites, toxicant in lipid droplets must move from the droplet into the freely dissolved phase in plasma before following concentration gradients into tissues. Theoretically this allows for the addition of therapeutic extracorporeal “exits” to the lipid shuttle, potentially mitigating toxicity through augmented clearance. Dialysis represents one such potential “exit”.

Dialysis presently plays an important role in the management of a limited number of intoxications [4]. Broader use of dialysis in toxicology is limited by two factors. Firstly, in many intoxications the amount of toxicant in blood available for dialysis is low relative to the total body load. This situation correlates with a large intrinsic Volume of distribution (Vd) of a toxicant, and we identify this problem as the *P*roblem of *V*olume of *D*istribution (PoVd). In general, dialysis is not useful for toxicants with a Vd greater than 2 L per kilogram [5]. The PoVd is theoretically amenable to manipulation of blood toxicant carriage with either introduced lipid emulsion or other intravascular drug binders. To successfully mitigate the PoVd, any such introduced binders would need to release toxicant at the dialytic membrane. This relates to the second problem of toxicant dialysis—that toxicant bound in blood (usually to plasma proteins) is generally not available to be exchanged into dialysate. This is quantitatively described by the percentage of toxicant bound to plasma proteins in blood. Dialysis is not considered beneficial for toxicants with protein binding greater than 85% [5]. We describe this as the *P*roblem *o*f *E*xtraction (PoE).

### Previous experiments investigating drug binding particles to augment dialysis

Mitigating PoE in dialysis using nanoparticles in dialysate has previously been investigated. As early as 1965 Shinaberger et al. [6] demonstrated lipid emulsion in peritoneal dialysate increased the dialysate:blood concentration ratio in glutethimide toxic dogs. Subsequent investigations have demonstrated PoE is mitigated for a number of drugs in a rat peritoneal dialysis model using pH gradient liposomes [7–9]. pH gradient liposomes are nanospheres of lipid bilayer with an acidic aqueous core, which entrap weakly basic cardiotoxins via ion trapping. When introduced into dialysate it is believed that pH gradient liposomes keep dialysate free drug concentrations low, which creates a concentration gradient favouring movement of drug from bound to free in blood, across the dialysis membrane to free in dialysate ending in drug entrapment within the pH gradient liposome nanoparticle.

Our group have previously triailled intravascular lipid emulsion and liposomes to mitigate PoVd without demonstrating effect in acute intravenous and enteral models of drug toxicity [10–12]. Harvey et al. demonstrated a 60% reduction in Vd and a tenfold increase in dialysate concentrations when lipid emulsion was used in both blood and dialysate in clomipramine toxic rabbits compared with 0.9% saline in blood and dialysate [13]. The design of this study did not permit attribution of the increased dialytic extraction to mitigating PoVd with intravenous lipid emulsion or PoE with intraperitoneal lipid emulsion. No other study has shown any signal that PoVd could be mitigated with an intravascular drug binder. A summary of studies in this area is given in Table 1.

**Table 1:** Summary of studies where nanoparticles were used to mitigate PoE ± PoVd

Author/year	Route of intoxication/intoxicants	Dialysis model	Strategy for PoVd	Strategy for PoE	Result
Shinaberger 1965 [6]	Intramuscular glutethimide in dogs	Peritoneal and low surface area/flux haemodialysis in dogs	Nil	Lipid emulsion as dialysate	fivefold increase in ratio blood:dialysate conc in PD No significant increase in haemodialysis extraction with LE (though low area/flux)
Forster et al. 2014 [7]	Variety of drugs	Peritoneal dialysis in rats	Nil	pH gradient liposomes in dialysate	Increased extraction of verapamil, propranolol, haloperidol and amitriptyline
Forster et al. 2012 [8]	Acute enteric verapamil	Peritoneal dialysis in rats	Nil	pH gradient liposomes in dialysate	80 fold increase in verapamil in dialysate; mitigation of verapamil induced haemodynamic compromise
Cave et al. 2022 [9]	Acute intravenous amitriptyline	Peritoneal dialysis in rats	Nil	pH gradient liposomes in dialysate	Total free amitrip perfusing peritoneum less than total in dialysate; protein bound amitrip extracted
Cave et al. 2019 [10]	Intravenous amitriptyline bolus	Peritoneal dialysis in rats	Intravenous liposomes which bound drug in membrane	pH gradient liposomes in dialysate	No increase in dialytic extraction of amitriptyline with IV liposomes
Chapman et al. 2015 [11]	Acute enteric amitriptyline	Peritoneal dialysis in rats	Intravenous lipid emulsion (ILE)	pH gradient liposomes in dialysate	No increase in dialytic extraction of amitriptyline with ILE
Cave 2019 [12]	Intravenous amitriptyline bolus	Peritoneal dialysis in rats	ILE	pH gradient liposomes in dialysate	No increase in dialytic extraction of amitriptyline with ILE
Harvey et al. 2009 [13]	Intravenous clomipramine bolus + infusion	Peritoneal dialysis in rabbits	ILE	Lipid emulsion as dialysate	60% reduction in Vd with ILE. IV + IP LE 10 times clomipramine concentration in dialysate compared with IV + IP saline

### Limitations of previous work

The expert body which makes recommendations on dialysis in intoxication proscribe dialysis in tricyclic antidepressant (TCA) poisoning [14]. With a volume of distribution (Vd) of 14 L per kilogram [14], reductions in Vd of the magnitude seen in previous experimental work would not bring about sufficient reduction in Vd to make TCA’s a rational target for dialysis [5]. Investigations seeking to mitigate PoE/PoVd using TCA’s, including the present work, are useful to explore concepts which may be applicable to other intoxications rather than leading to the use of dialysis in TCA intoxication.

Another limitation in past experimental work which has explored simultaneous solutions to the PoVd and PoE is that intravascular particles were introduced outside a state of pharmacokinetic equilibrium. This meant the intravascular binder reduced freely dissolved toxicant concentration in blood [12], tending to reduce the diffusion of toxicant into dialysate. We sought to mitigate this limitation by introducing lipid emulsion after longer term oral exposure to amitriptyline when tissues had reached steady state concentrations. We expected that saturation of tissue beds at steady state would mitigate the effect of intravascular lipid emulsion on free blood concentrations.

### Hypothesis tested in this experiment

**Fig. 1:**
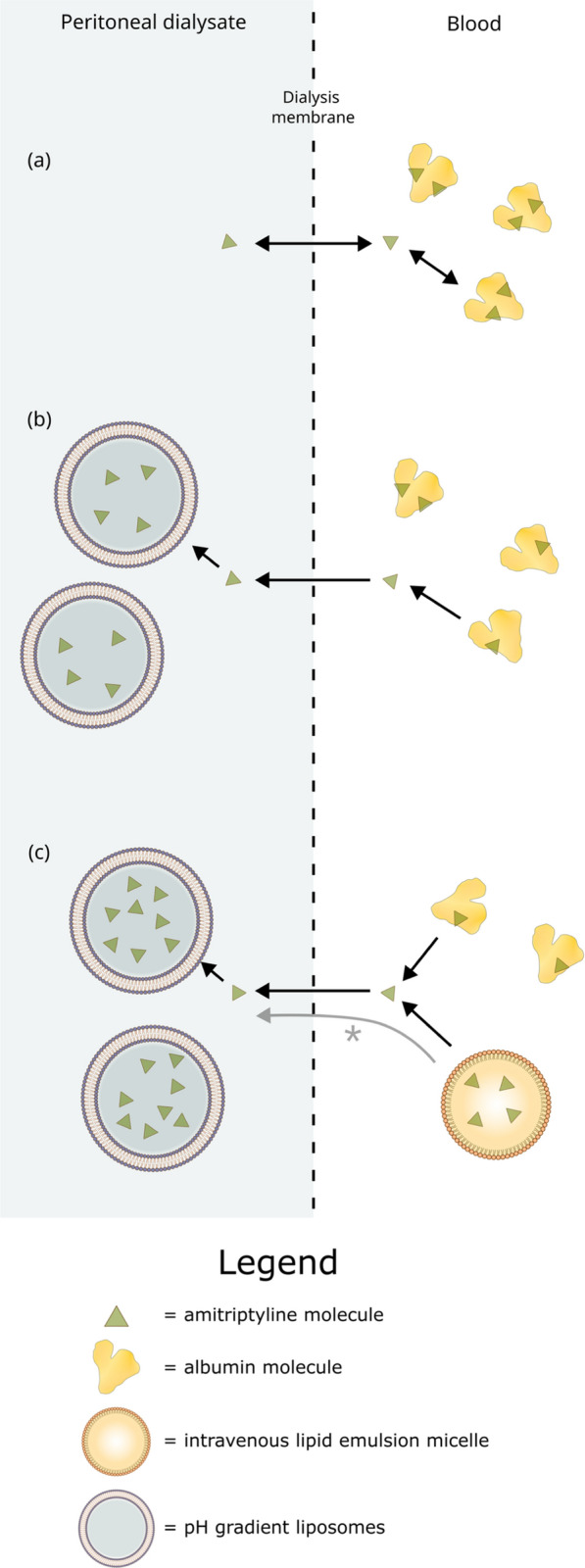
**a** Representing the problems of PoVd, with relatively low total drug concentration and PoE, with only the very low free concentration able to equilibrate with dialysate. **b** Representing a pH gradient liposome in dialysate, which have been shown to experimentally to mitigate PoE [7–9]. Capture of drug in liposomes in dialysate keeps free drug concentration in dialysate low. Drug moves from the free phase in blood down a concentration gradient into dialysate, and from albumin into the free phase in blood. **c** Representing the addition of lipid emulsion particles to the blood at steady state. It is presumed that at steady state the effect of lipid emulsion on free drug concentrations in blood will be small. The *arrow* marked * represents our study hypothesis—will drug in lipid emulsion particles in blood be available for extraction into liposome augmented dialysate?

We tested the hypothesis that the addition of intravenous lipid emulsion would increase extraction of amitriptyline into pH gradient liposome augmented peritoneal dialysate in chronically amitriptyline exposed rats. The hypothesis for our experiment is outlined graphically in Fig. 1.

## Methods

### Liposome preparation and validation

We prepared liposomes in 20 mL batches in our facility in Wellington New Zealand one week prior to the experiment using the thin film method, as previously described [15]. In brief, 100 mg of pure lipids in molar ratios 54% dipalmitoylphosphatidylcholine [DPPC]; 45% cholesterol and 1% 1,2-distearoyl-sn-glycero-3-phosphoethanolamine-N-[methoxy(polyethylene glycol)-2000] [DSPEmPEG 2000] (Merck, Darmstadt, Germany) were dissolved in 20 mL 99% ethanol/1% methanol (Pure Nature, Auckland, New Zealand). The organic solvent was evaporated off in a rotating spherical flask suspended in a water bath at 40 °C while a stream of pure nitrogen was passed over the solution, until a thin film formed on walls of the flask. Once removed from the water bath, the stream of nitrogen was continued for another 2 h to ensure that the thin film was completely dried.

We prepared a solution of 250 mM citric acid by adding 9.6 gm of citric acid (Pure Nature, Auckland, New Zealand) to 200 ml sterile water (Fresenius Kabi, Bad Homburg, Germany). pH of this solution was measured at 2.2. 20 mL of this solution was added to each thin film and the flask was then rotated in a bath ultrasound at 40 kHz until all visible film was suspended. The flask was then placed upright in the ultrasound bath for 10 further minutes to ensure all lipid was fully suspended.

Liposome size was measured using a Malvern mastersizer 3000 (Malvern Panalytical, Malvern, England).

### In vivo experiment

#### Location

We undertook this experiment in the Small Animals Colony of the Ruakura Agresearch facility in Hamilton, New Zealand.

#### Ethical approval

All animal protocols for this experiment were approved by the Agresearch Ruakura Animal Ethics Committee. The project approval number was 2339.

#### Animals

Animals were kept in single gender enclosures with no chance of pregnancy. Twelve-hour light–dark cycles (lights on/off at 07:00/19:00 h) and climate control were maintained. We used 20 female Sprague–Dawley rats for this experiment. Four were used for initial dose ranging experiments and 16 for the full study protocol.

#### Amitriptyline dose ranging experiments

In order to create a steady state model we added amitriptyline to the drinking water of 4 rats for 7 days. This duration was chosen as it represents greater than four half-lives of amitriptyline in the rat, the time taken to reach a presumptive steady state [16, 17]. At the end of seven days exposure we took a tail vein blood sample under isoflurane anaesthesia. Animals were returned to a group enclosure on recovery. There was a washout period of at least 2 weeks amitriptyline free before repeating at a higher dose if needed. Before a higher dose of amitriptyline was used water intake during the dosing period was analysed, and dose was not subsequently increased if there was a 10% or greater reduction in water intake from baseline. The tail vein blood sample was sent for amitriptyline concentration measurement. Once we identified a dosing regimen that permitted study and did not reduce water intake (estimated 50 mg/kg/day amitriptyline), the 4 animals used in this phase were euthanized according to institutional protocols.

#### Dialysis experiment

We used 16 animals aged 24–28 weeks in this phase of the experiment. At commencement of the study protocol, animals were sedated with ketamine at 50 mg/kg (Mayne Pharma Ltd., Auckland, New Zealand), and xylazine at 4 mg/kg (Bayer HealthCare, Leverkusen, Germany) via single intraperitoneal injection. We then placed animals on a warming board at 38.5 °C. An intravenous (IV) cannula was placed in the tail vein using a needle over catheter technique (24 G × 0.75 in Insyte, BD, Switzerland). 1% lignocaine was instilled in the anterior neck and following dissection a tracheostomy tube (14 G × 1.77 in Insyte, BD, Switzerland) was placed under direct vision. Mechanical ventilation using a small animal ventilator (Inspira ASV, Harvard Apparatus, USA) was then undertaken with 100% oxygen as the driving gas admixed with 2% isoflurane (Merial, Auckland, New Zealand) via a vaporizer (Somnosuite Small Animal Anesthesia System, Kent Scientific Corporation, Torrington, USA) to maintain anaesthesia.

We placed a carotid arterial line under direct vision using a catheter over needle technique, tying off the hub of the catheter and the artery a small distance cephalad to insertion (24 G × 0.75 in Insyte, BD, Switzerland). The arterial catheter was used for blood sampling and to monitor arterial pressure and heart rate via the arterial pressure waveform (BP Amp, AD Instruments, Bella Vista, Australia). Anaesthesia, mechanical ventilation and blood pressure monitoring were continued for the duration of the experiment. Haemodynamic metrics were recorded on a standardized template at predetermined time points in the experiment. We monitored depth of anaesthesia at 5 min intervals using paw squeeze and corneal reflex.

At the completion of instrumentation there was a stabilization period of 5 min for each animal. After this a baseline blood sample was taken to measure amitriptyline concentration (nominally at 0 min, the start of the experiment). All blood samples taken during the experiment were 0.5  mL in volume. Animals were randomized into 2 groups of 8 to receive either saline control or intravenous lipid emulsion. The saline control animals received 5ml of 0.9% saline (Baxter Pharmaceuticals, Toongabbie, Australia) as a single slow intravenous injection at the start of the experiment. The lipid emulsion animals received 1ml of 20% Intralipid® (Fresenius Kabi, Bad Homburg, Germany) at the start of the experiment followed by an infusion at 0.07 mL per minute. We used this dose of lipid emulsion as we calculated it would maintain 1% lipaemia in a 300-g rat with an assumed blood volume of 20mL and a half-life of lipid droplets of 10 min [18]. The difference in dosing for the two intravenous agents was based on an expected difference in intravascular half-life [19].

A second blood sample was taken 10 min after the first blood sample.

We then centrifuged 10 × 1 mL tubes of 5 mg/mL pH gradient DPPC/cholesterol liposome suspension at 17,500 G for 10 min to form a liposome pellet at the base of the test tube. At 46 min post the first blood sample we aliquoted off remaining citrate and resuspended liposomes in Hemosol (Baxter, Deerfield, Illinois, USA). We then added resuspended liposomes to final volume of 20 mL, to make a dialysate containing 50 mg of pH gradient DPPC/cholesterol liposomes suspended in 20 mL of Hemosol.

As this protocol ran with a single operator the pre dialysis blood sample was taken 5 min prior to commencement of dialysis (T45) for amitriptyline concentration. We regarded this as the blood concentration at the commencement of dialysis for analysis.

At 50 min we injected 20 ml of the dialysate described above into the peritoneum. The dialysate was manually agitated at one-minute intervals until the end of the experiment.

At 60 min we euthanized all animals by injection of pentobarbitone in the tail vein. This resulted in rapid demise with universal cessation of the circulation as assessed by the arterial line trace within seconds.

After demise, we sampled blood and peritoneal fluid for amitriptyline concentration measurement.

Average blood amitriptyline concentration during dialysis was taken as the average of the blood concentrations at 45 and 60 min.

#### ERest

In previous work we have estimated peritoneal blood flow over the dwell and divided the amount extracted into dialysate by the estimated amount of amitriptyline which perfused the peritoneal membrane during the dwell [10, 11]. We defined this as the estimated extraction ration (ERest). In this experiment we estimated peritoneal blood flow at 2 mL/min [20]. A 10-min dwell time with a 20 mL dialysate volume meant ERest was calculated to be the ratio of dialysate to average blood concentration during peritoneal dwell.

#### Amitriptyline concentration measurement

Amitriptyline concentrations were measured using a liquid chromatography–mass spectrometry method (Sciex 3200 MSD Aglient 1200 series LS, Sciex [Redwood City, USA]) with isotope matched internal standards. The whole blood sample was prepared by a protein crash with acetonitrile, followed by centrifugation after which an aliquot was presented to the instrument for analysis. A four-point matrix-matched standard curve was used to calibrate the analyser for each sequence with two levels of quality control.

#### Statistical analysis

We powered this experiment for a 50% increase in blood and peritoneal dialysate concentration of amitriptyline with the intravenous lipid emulsion using Student’s t test. We used a mean blood concentration 200 nmol/L and standard deviation of 50 nmol/L in powering the study [21]. With a standard deviation of 50nmol/L and a true difference of 100 nmol/L, we achieved > 80% power that an effect would be observed on blood concentrations with 8 subjects in each group.

The ratio of standard deviation to expected effect size is the same for peritoneal dialysate as for blood [7], giving the same power with 8 subjects.

Data were analysed using GraphPad Prism®. Mann–Whitney, one-way ANOVA and two-way repeat measures ANOVA were used as appropriate.

## Results

All values are shown as median (interquartile range) unless otherwise stated.

### Liposome size

Liposomes had a median particle size of 8.86 (5.28–14.90) micrometres.

### Baseline variables

There were no statistically significant differences between groups for baseline weight, pulse or mean arterial pressure. Baseline physiologic variables are shown in Table 2.

**Table 2:** Baseline variables

	Control	Lipid	*p* value
Weight (grams)	286 (284–290)	292 (286–301)	0.37
Baseline pulse (bpm)	227 (224–227)	212 (197–240)	0.35
Baseline MAP (mmHg)	64 (56–67)	61 (49–69)	0.87

There was no significant change in MAP over time for the experimental period for either group; controls *p* = 0.08, Lipid *p* = 0.28, one-way ANOVA. Pulse rate decreased over time in both groups (one-way ANOVA, *p*, 0.01 for both groups). Treatment allocation did not significantly alter the decrease in pulse over time (*p* = 0.42, 2-way repeated measures ANOVA).

### Haemodynamic variables during peritoneal dialysis

There were no statistically significant differences for pulse rate. The mean of MAP during the dialysate dwell was higher for IL rats, but this difference did not reach statistical significance. These variables are shown in Table 3.

**Table 3:** Average haemodynamic variables during dialysis dwell

	Control	Lipid	*p* value
Pulse during dwell (bpm)	190 (185–204)	198 (174–206)	0.91
MAP during dwell (mmHg)	49 (44–55)	61 (56–63)	0.06

### Blood concentrations of amitriptyline

There were no statistically significant differences within groups for blood concentrations of amitriptyline at time points 0, 10 min or prior to dialysis (*p* = 0.15 control, lipid *p* = 0.39, one-way ANOVA).

**Fig. 2:**
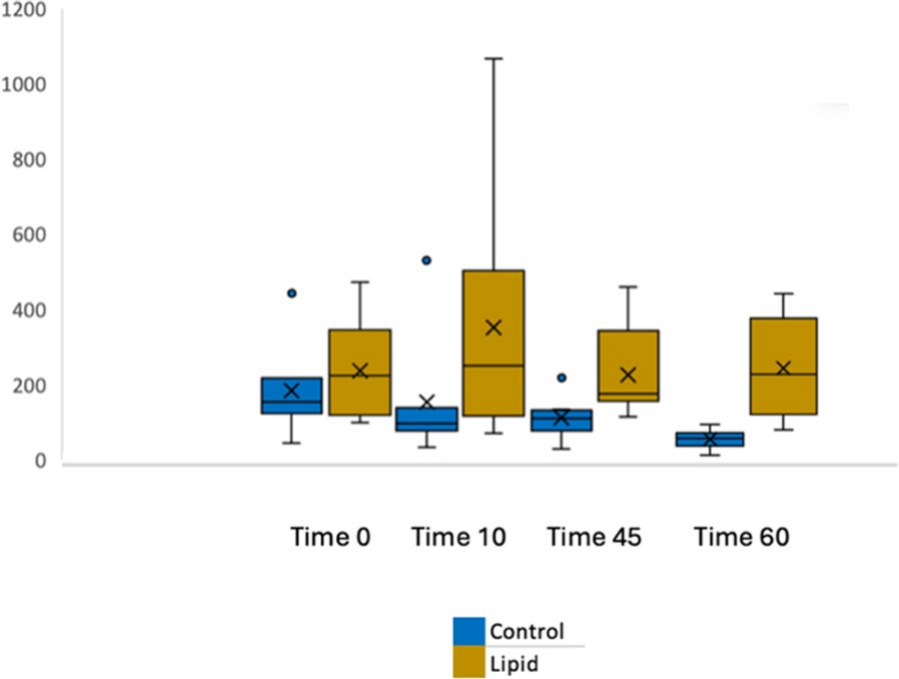
Box and whisker plot, amitriptyline blood concentration over time (nmol/L)

**Fig. 3:**
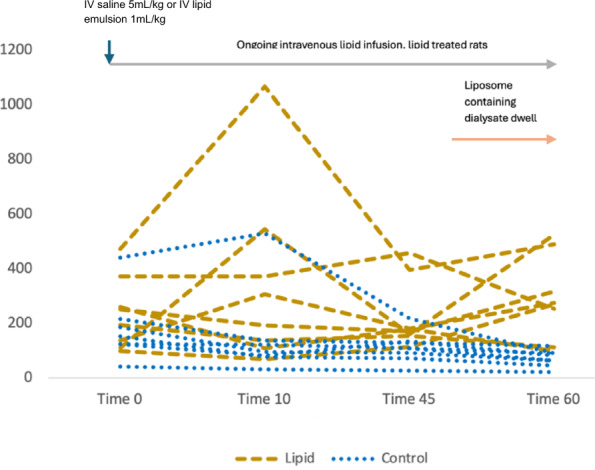
blood amitriptyline concentration over time, individual subjects (nmoll/L)

Box and whisker plots for blood concentration over time are shown in Fig. 2. Blood amitriptyline concentrations at T0 were not different between groups; control 153 (125–201) nmol/L, lipid 223 (130–287) nmol/L, *p* = 0.45. Time weighted individual subject mean blood amitriptyline concentrations from Time 0 to Time 60 were higher in the lipid group than the control group; control 104 (87–125) nmol/L, lipid 219 (148–357) nmol/L, *p* = 0.03. A significant effect was seen when time weighted average concentration was analysed for both the time periods prior to (Time 0 to Time 45) and during dialysis (Time 45 to Time 60). Blood amitriptyline concentrations for each subject over the course of the experiment are shown in Fig. 3.

Blood amitriptyline concentrations for each subject over the course of the experiment are shown in Fig. 3.

### Dialysate results

**Fig. 4:**
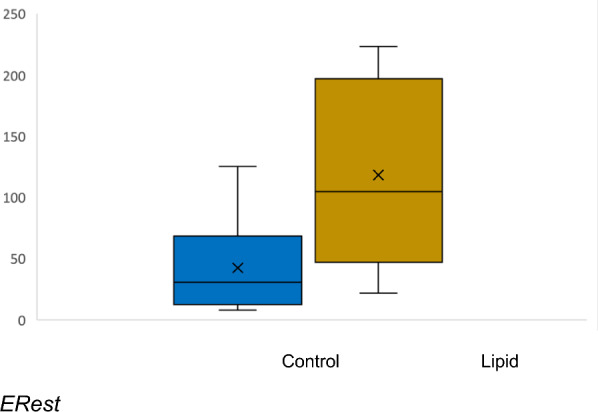
Concentration of amitriptyline in 20 mL dialysate, nmol/L

During the 10 min peritoneal dialysate dwell a greater amount of amitriptyline was extracted into the 20 mL of dialysate in the lipid emulsion group than the control group; control 31(14–52) nmol/L, lipid 105 (62–185) nmol/L, *p* = 0.03. Results are shown graphically in Fig. 4.

### ERest

There was no significant difference in ERest between groups. ERest was 0.54 (0.37–0.92) for the lipid group and 0.34 (0.2–0.5) for controls, *p* = 0.59.

## Discussion

To our knowledge, these results represent the first experimental support for utilizing drug binding particles in both blood and dialysate to mitigate the PoVd and PoE simultaneously. Although amitriptyline is an unlikely target for future clinical application, our results support more work being done using this conceptual framework to work towards potentially increasing the scope of dialysis in intoxication.

The primary endpoint to which our study was powered was positive—dialysate amitriptyline concentrations were greater in the intravenous lipid group. The difference in time weighted blood concentration for lipid and control subjects correlates to a reduction in Vd of 53%, consistent with previous work showing a reduction in Vd of 60% [13]. There was also no significant difference in ERest. Taken together these findings suggest amitriptyline contained in lipid emulsion droplets in blood was available for dialysis in our model. Although many problems remain to be solved, protein bound toxicants of similar lipophilicity to amitriptyline with Vd’s up to 5 L/kg could conceivably be bought into a dialysable range with the simultaneous use of appropriate toxicant binders in blood and dialysate. Future work may focus on protein bound toxicants such as verapamil and diltiazem with reported Vd in this range [22, 23].

### Limitations

Amitriptyline has been used in our work for its pharmacokinetic properties and availability of an assay for the purposes of investigation of concept. There is low potential to bring the volume of distribution and capacity for extraction from blood for amitriptyline into a range that would be potentially clinically useful.

Our results at pharmacokinetic steady state are consistent with toxicant in lipid droplets being available for exchange across a dialysis membrane. This contrasts with previous work when intravascular binders were given very shortly after administration of toxicant. Absence of effect in these models may have been because free toxicant concentration was reduced [12]. Neither steady state nor intravenous bolus administration replicate the balance between absorption and metabolism in a critically ill patient many hours after clinically important overdose. Of note, a three-hour pH gradient liposome augmented dialysate dwell has been shown to reduce toxicity in rats with enteric verapamil overdose when given one hour after dosing [7]. Also important is that solutions to PoVd may paradoxically increase toxicity if instituted during drug absorption by increasing gut uptake of drug [24, 25]. Potential clinical application of any simultaneous PoVd/PoE manipulation may be best considered for the patient who has largely absorbed and distributed toxicant and remains unstable.

Peritoneal blood flow was not measured in this experiment and reduced peritoneal blood flow could have contributed to the lower amitriptyline dialysate concentration in the control group. A nearly equal volume of fluid was given to both groups, although in the saline controls this was given at the commencement of the protocol, and animals may have reacted differently to the 1.5 ml (estimated 7.5%) blood loss associated with the three blood samples taken prior to the end of the dialysate dwell. Additionally, lipid emulsion when infused outside the setting of toxicity has been associated with increases in cardiac output [26]. It is possible these factors led both to a reduction in blood amitriptyline concentration prior to peritoneal dialysis and/or a lower ERest in control animals that was beyond the power of this experiment to demonstrate. However, differences in ERest were not statistically significant and the effects on amitriptyline dialysate concentration seen with intravascular lipid in the present experiment are of simlar magnitude to previous in vivo work in rabbits [13]. Future in vitro haemodialysis models would be useful to control for uncontrolled haemodynamic parameters in this experiment.

Blood amitriptyline concentrations in our experiment were lower than those associated with clinical toxicity. Although the ERest in controls was similar to previous models where toxic concentrations were seen [10], it is possible the effects on dialytic extraction would reduce at higher blood concentrations.

Peritoneal dialysis is not used acutely in toxicology and there is no realistic prospect of its use in the future. We utilized peritoneal dialysis as it is the model we have experience with, and it is methodologically simple. Issues with the potential unmeasured confounder of peritoneal blood-flow and the potential for different behaviour of the peritoneal membrane and other dialysis membranes used in any potential clinical application represent limitations for this experiment.

Intravenous lipid emulsions are known to interfere with extracorporeal circuits. It is possible that heparin used in anticoagulation of the circuits may play a role in this interference, as it is known to flocculate lipid emulsions [27]. Solving the problem of lipid emulsion interference with extracorporeal circuits or use of another reversible intravascular drug binder to solve the PoVd are significant future considerations in this area.

We attempted to measure unbound blood concentrations of amitriptyline in this experiment. The technique we used to measure free concentrations was centrifugal pharesis across a membrane with a protein free solution forming in the test tube beneath the pharesis membrane after centrifugation. All but one of the measurements came back as zero nmol/L, which we attributed to amitriptyline adsorption to the pharesis membrane at the blood amitriptyline concentrations seen in our study. Given this, the assumption that intravenous lipid emulsion did not affect free drug concentrations in blood in our experiment remained untested.

### Future experimental directions

Future experimental work may focus on other reversible intravascular binders to solve the PoVd using toxicants such as verapamil and diltiazem with lower Vd’s more likely to be reduced into a dialysable range. An important feature of any potential solution to the PoVd in this context is compatibility with anticoagulation and dialysis membranes.

pH gradient liposomes are a promising solution to the PoE. A potential avenue in intoxication may be the use of such liposomes in continuous veno-veno haemodialysis (CVVH). Clinical trials of the use of liposomes in peritoneal dialysate to manage the hyperammonaemia of liver failure are currently active [28]. pH gradient liposomes in CVVH dialysate could maintain low dialysate toxicant concentration independent of dialysate flow rate and convert the factor which limits CVVH drug clearance from dialysate flow to bloodflow.

Strategies to displace bound toxicant from Sudlow binding sites such as infusing Ibuprofen [29] in the afferent arm of an extracorporeal circuit is another avenue for future work.

## Conclusion

To the authors knowledge this is the first successful experiment combining strategies to both decrease the volume of distribution using an intravascular toxicant binding nanoparticle with a toxicant binding particle in dialysate. Future work may use the framework of this experiment with the aim of increasing the number of target molecules for dialysis.

## Data Availability

Data will be made available on reasonable request.
